# Case Report: Kinetics and durability of humoral and cellular response of SARS-CoV-2 messenger RNA vaccine in a lung and kidney transplant recipient

**DOI:** 10.3389/fimmu.2023.1207638

**Published:** 2023-07-03

**Authors:** James Long, Mithil Soni, Pawel Muranski, Maureen J. Miller, Cathleen Conry-Cantilena, Valeria De Giorgi

**Affiliations:** ^1^ Infectious Diseases Section, Department of Transfusion Medicine, National Institutes of Health Clinical Center, Bethesda, MD, United States; ^2^ Columbia Center for Translational Immunology, Herbert Irving Comprehensive Cancer Center, Columbia University Irving Medical Center, New York, NY, United States

**Keywords:** COVID-19, SARS-CoV-2 vaccine, humoral immunity, cellular immunity, immunocompromised, solid organ transplant, case report

## Abstract

We present a case report of a 63-year-old female health care worker who is 15 years status post double lung transplant and six years status post living related donor kidney transplant who is healthy on a chronic immunosuppression regimen including prednisone, mycophenolate, and tacrolimus who received the SARS-CoV-2 mRNA vaccine (Pfizer-BioNTech BNT162b2) primary series and had poor initial humoral response to the COVID-19 mRNA vaccine, then demonstrated a robust, sustained immune response against S1 and S2 antigens for over seven months after receiving the recommended vaccine doses, including booster dose, without developing COVID-19 or other serious adverse events. Her immune response to vaccination indicates effective formation of anti-spike T cell memory despite chronic immunosuppression. This case report provides a comprehensive characterization of her immune response to this SARS-CoV-2 vaccination series. As vaccine effectiveness data is updated, and as better understanding of immune response including hybrid immunity emerges, these findings may reassure that recipients of SOTs may be capable of durable immune responses to emerging variants of SARS-CoV-2.

## 1 Introduction

Since the discovery of the SARS-CoV-2 virus in 2019, the virus has developed numerous mutations leading to new variants with the potential for significant impact on public health during the COVID-19 pandemic and beyond. On November 26, 2021, the WHO designated Omicron (B.1.1.529) as the most recent variant of concern. By January 2022, Omicron was the predominant variant in the United States (1). This variant demonstrates over 30 mutations in the spike protein, 15 of which occur in the receptor-binding domain. Preliminary studies have shown that these changes may allow Omicron to evade neutralizing antibodies produced from both natural infection and immunization, resulting in breakthrough infections (2).

Reports of immunogenicity of the SARS-CoV-2 messenger RNA (mRNA) vaccine show that solid organ transplant (SOT) recipients are less likely to form antibodies after two doses to the S1 domain of the SARS-CoV-2 spike protein compared to people without immunocompromise, as measured by enzyme immunoassays (3). A third booster dose was recommended. More recently, a fourth dose was suggested to improve response in immunocompromised individuals (4).

Evidence demonstrating the long-term durability of the mRNA vaccine-induced immune response against SARS-CoV-2 in people with SOT is limited. Early data suggest that antibody titers wane after three months following a third dose (5). Sero-responders have demonstrated a cellular immune response early after a third vaccine dose, but little is known about the durability of T cell response over many months (6).

We present a comprehensive characterization of the immune response of a 63-year-old female health care worker who is 15 years status post double lung transplant and six years status post living related donor kidney transplant and healthy on a chronic immunosuppression regimen to this SARS-CoV-2 vaccination series. She received the SARS-CoV-2 mRNA vaccine (Pfizer-BioNTech BNT162b2) primary series and booster dose and had poor initial humoral response to the COVID-19 mRNA vaccine, then demonstrated a robust, sustained immune response against S1 and S2 antigens for over seven months after receiving the recommended vaccination doses.

## 2 Case description

A 63-year-old female health care worker who is 15 years status post double lung transplant and six years status post living related donor kidney transplant who is healthy on a chronic immunosuppression regimen including prednisone, mycophenolate, and tacrolimus. She received the SARS-CoV-2 mRNA vaccine (Pfizer-BioNTech BNT162b2) primary series on December 28, 2020 and January 14, 2021, with a booster dose on August 24, 2021.

## 3 Timeline of episodes of care

A timeline with relevant data from the episodes of care (vaccinations) shows the patient’s antibody generation after the primary COVID-19 vaccination series and booster doses (**Table 1**). We describe the antibody assays and measurements in more detail in the next section (Diagnostic Assessment and Therapeutic Intervention).

**Table 1:** Timeline with relevant data from the episodes of care (SARS-CoV-2 mRNA vaccine (Pfizer-BioNTech BNT162b2) vaccinations, including primary series and booster doses) and tests performed.

Timeline	SARS-CoV-2 mRNA vaccine (Pfizer-BioNTech BNT162b2)	Tests Performed
December 28, 2020	Primary Series	*(no test – vaccination)*
January 14, 2021	Primary Series	*(no test – vaccination)*
February 17, 2021		VITROS Anti–SARS-CoV-2 Total and IgG; FRNT*
March 18, 2021		VITROS Anti–SARS-CoV-2 Total and IgG; FRNT
May 5, 2021		VITROS Anti–SARS-CoV-2 Total and IgG; FRNT
June 9, 2021		VITROS Anti–SARS-CoV-2 Total and IgG; FRNT
July 23, 2021		VITROS Anti–SARS-CoV-2 Total and IgG; FRNT; T-cell response
August 20, 2021		VITROS Anti–SARS-CoV-2 Total and IgG; FRNT
August 24, 2021	Booster	*(no test – vaccination)*
September 13, 2021		VITROS Anti–SARS-CoV-2 Total and IgG; FRNT
October 28, 2021		VITROS Anti–SARS-CoV-2 Total and IgG; FRNT
November 2, 2021		T-cell response
December 3, 2021		VITROS Anti–SARS-CoV-2 Total and IgG; FRNT
January 21, 2022		VITROS Anti–SARS-CoV-2 Total and IgG; FRNT
February 8, 2022		VITROS Anti–SARS-CoV-2 Total and IgG; FRNT; T-cell response
April 29, 2022		VITROS Anti–SARS-CoV-2 Total and IgG; FRNT

*FRNT, fluorescence reduction neutralization assay.

## 4 Diagnostic assessment and therapeutic intervention

### 4.1 Diagnostic assessment

The patient was a healthy outpatient on a chronic immunosuppression regimen who received regular clinical monitoring during the COVID-19 pandemic. All of the antibody and T cell data presented in the paper were collected before this additional boost and mAb therapy.

### 4.2 Therapeutic intervention

The patient received the SARS-CoV-2 mRNA vaccine (Pfizer-BioNTech BNT162b2) primary series on December 28, 2020 and January 14, 2021, with a booster dose on August 24, 2021.

To monitor the patient’s immune response to the vaccinations, the health care worker’s anti-SARS-CoV-2 serum antibody level was tested every one to two months from February 2021 to April 2022. Total (including IgA, IgM, IgG) and IgG antibodies against the SARS-CoV-2 spike protein were assessed using the Ortho-Clinical Diagnostics anti-SARS-CoV-2 assay qualitative chemiluminescence immunoassay (ChLIA), performed on the VITROS 3600 automated immunoassay analyzer (7). Anti-SARS-CoV-2 Total N Antibody test was also performed. Antibody levels are expressed as the ratio of the sample signal to a calibrator-assigned cutoff signal (S/Co). The assays for determining neutralizing titers were performed with authentic SARS-CoV-2 (2019-nCoV/USA-WA1-A12/2020 from the US Centers for Disease Control and Prevention, Atlanta, GA) at the NIH-NIAID Integrated Research Facility at Fort Detrick, MD, using an FRNA as described by Holbrook et al. (8).

T-cell response was analyzed at three time points: 1) at 6 months post full vaccination, 2) at 2 months post booster (around 10 months post full vaccination) and 3) at around 5 months post booster shot (13 months post full vaccination). To further test if vaccination-induced T cell immunity cross-reacts against SARS-CoV2 variants, we challenged ancestral spike primed T cells with S1 and S2 counterpart of omicron variant. We also examined T-cell response against other structural SCoV2 antigens – Membrane (M) and Nucleocapsid (N) protein. To precisely gauge the immune response of the subject, we used microscale priming/expansion strategy, a highly sensitive method capable of unequivocal detection of pre-established T cell responses against viral antigens even when the frequency of the memory T cells is at the background level. In brief, cryopreserved PBMCs were pulsed with overlapping 15-aminoacid long peptide libraries spanning the full length of S1, S2, M and NP antigens (final concentration of 1 mg/mL) and expanded for 14 days in AIM V medium (Thermo Fisher Scientific, Waltham, MA, USA) supplemented with inactivated 5% human serum, IL-7 at 10 ng/mL and IL-2 at 30 IU/mL (PeproTech, Rocky Hill, NJ, USA). Final cultures were restimulated with indicated peptide mixes for 6 h in the presence of brefeldin A and monensin A (BD Biosciences, San Jose, CA, USA) per the manufacturer’s instructions followed by intracellular cytokine staining and flow cytometry to quantify antigen-specific T cell response (9). Gating strategy for the analyzing antigen specific T cells has been shown in **Supplementary Figure 1A**. All plots were generated using GraphPad Prism version 8.4.3.

### 4.3 Follow-up and primary outcomes

**Figure 1:**
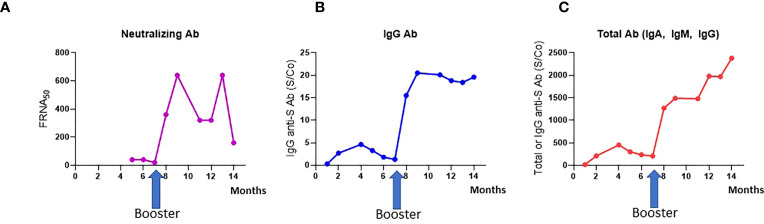
Antibody generation after COVID-19 vaccination. **(A)** Neutralizing Ab titers; **(B)** IgG antibody against the Spike protein (S); **(C)** Total antibody (IgA, IgM, IgG), measured over time after three vaccine doses.

The health care worker had detectable total but negative IgG antibodies four weeks after the second dose (S/Co=15.1 and S/Co= 0.3 respectively). IgG antibodies were detected two months after the second dose (S/Co= 2.69). Total, IgG and neutralizing antibodies level increase from S/Co= 207, S/Co= 1.3 and 1:20 before the booster to S/Co=1270, S/Co=15.5 and 1:360 one month after the booster respectively. She was still seropositive eight months after the booster dose with total, IgG and neutralizing antibodies level of S/Co= 2380, S/Co= 19.6 and 1:160 respectively (**Figures 1A–C**). Antibodies to the nucleocapsid protein were not detected (data not shown).

**Figure 2:**
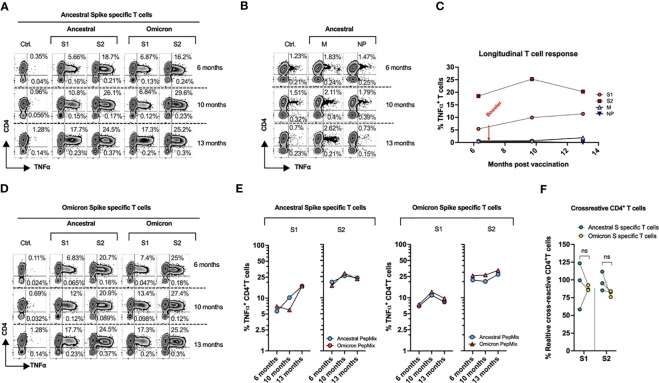
Kinetics of T-Cell Response post SARS-CoV-2 mRNA vaccine. PBMCs from the subject collected at three different time points (189, 292, and 390 days post vaccination), were primed with the ancestral or omicron spike peptide library (S1 & S2), cultured for 14 days and tested against the indicated antigens. **(A)** Ancestral spike primed T cells were stimulated with S1 and S2 antigens from either ancestral or omicron spike followed by cytokine release assay. **(B)** Cytokine release assay of cultures primed with M and NP peptide and tested against cognate antigens post 14-day expansion. **(C)** Longitudinal T cell response against ancestral SARS-CoV2. **(D)** Omicron spike primed T cells were stimulated with S1 and S2 antigens from either ancestral or omicron spike followed by cytokine release assay. **(E)** Cross-reactivity of ancestral (Left) and omicron (Right) spike-specific T cells. **(F)** Percentage of cross-reactive cells among total reactive cells in both ancestral and omicron spike primed cultures.

A T-cell response against S1 and S2 peptide pool was observed after two doses of vaccination (**Figure 2A**), as previously documented in SCoV2 infected and/or exposed individuals who mounted a robust T cell response without seroconversion (10). Third vaccination dose further enhanced the reactivity against these antigens, with up to 17.7% and 24.5% of T cells recognizing S1 and S2 antigens respectively.

The ancestral-spike specific T cells were able to mount a response against omicron variant, with similar reactivity to that of cognate antigens at each tested timepoint (**Figure 2A**). Although the subject has not experienced documented COVID-19 illness, a degree of reactivity was seen against M protein. No response was elicited against NP antigens (**Figure 2B**).

Therefore, we examined response against human coronavirus alpha-hCoVs (229E and NL63) and beta- hCoVs OC43 and HKU1. A robust T-cell response was observed against hCoVs with four antigens from α-hCoV-NL63 and three out of four antigens of α-hCoV-229E were recognized, whereas a minimal reactivity was observed against both β-hCoVs (**Figure S1**).

Overall, she mounted a robust response against S1 and S2 antigens which persisted even after 13 months (**Figure 2C**), indicating an effective formation of anti-spike T cell memory despite chronic immunosuppression.

### 4.3 Secondary outcomes

To further examine vaccination induced response to omicron variant, we primed PBMCs from subject with full length omicron S1 and S2 peptide libraries. Day 14 cultures were re-challenged with cognate (omicron) antigens and with ancestral S1 and S2 peptide pools. We observed reciprocal cross-reactivity with Omicron S1/S2-induced T cells showing potent reactivity against ancestral counterparts (**Figures 2D, E**) at each analyzed timepoint. Both cultures did not show statistically significant difference in % cross-reactive cells among total reactive T cells (**Figure 2F**). Overall, these observations indicate that vaccination may provide some immune response against omicron variant.

**Figure 3:**
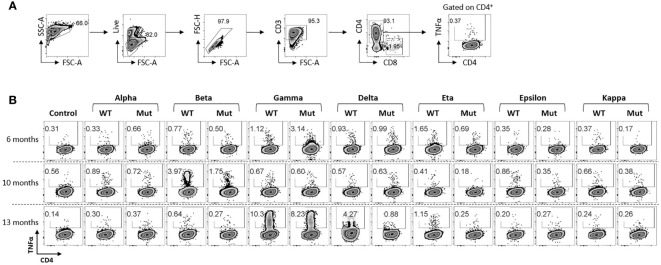
Response of ancestral spike-specific T-cells against various SARS-CoV2 variants: **(A)** Gating strategy used for the analysis of antigen reactive T cells. **(B)** Ancestral spike primed T cells were expanded for 14 days and challenged with indicated peptide libraries of variants of SCoV2. Each variant library consisted of peptides spanning mutated regions only. Dot-plots shows antigen-specific CD4^+^ T cell response.

We also examined the patient’s post-vaccination immune response against other known variants of concerns (VOCs) and variants of interests (VOIs), challenging ancestral spike specific T cells from all three time points with seven SARS-CoV-2 variants. Most of the variants that showed response against WT-epitope also showed response against mutant epitope, although to a lesser extent in some instances (**Figure 3**). Strong reactivity was observed against beta variant post booster dose (Draw 2, 10 months post vaccination) which was lost in draw 3 (13 months post vaccination), while a strong response was observed against gamma variant in draw 3. On the contrary, reactivity to delta variant was lost 390 days post vaccination. However, variant pools consisted of limited epitopes spanning only divergent regions (**Table S1**) and therefore represent only a minor portion of the overall T cell immunity against the full-length spike proteins. Total anti-spike T cell reactivity induced by vaccination (**Supplementary Figure 1**), potentially reflecting the broad T cell immunity against SARS-CoV-2 which targets the full length of the immunodominant viral proteins, is more comprehensive.

## 5 Discussion

This case report describes a 63-year-old female health care worker on a chronic immunosuppression regimen status post SOTs who responded to a SARS-CoV-2 mRNA vaccine (Pfizer-BioNTech BNT162b2) primary series and booster dose without adverse events. Antigen-specific T cell memory was readily detected 13 months post vaccination. Robust anti-spike T cell immunity emerged despite poor humoral responses after the initial course of vaccination. Sustained levels of IgG and total antibodies were observed after the third dose of mRNA vaccine at 8 months post vaccination. Overall, these observations indicate that the antibody production patterns observed in this subject after full vaccination suggest a partial or better immune response. against several variants of SARS-CoV-2. We show T cell responses in both CD4 and CD8 compartments in an unbiased way. There was evidence of T-cell response against S1 and S2 peptide pool after two doses of vaccination, as previously documented in SCoV2 infected and/or exposed individuals who mounted a robust T cell response without seroconversion (10). A third vaccination dose further enhanced the reactivity against these antigens. Speculations about possible mechanisms of this response are described elsewhere in the literature (11–13), but the real importance of the T cell responses is unknown.

Our subject has a complex immunologic history and may have idiosyncratic or previously undescribed immune responses compared to immune responses observed in other sero-responders status post SOT. A humoral response has been reported in SOT patients who received third doses of mRNA vaccine in recipients of kidney (14–16), lung (17), liver (18, 19) and heart (20, 21), and in one matched control study from Denmark matching healthy, vaccinated patients against fully vaccinated patients with histories of SOT (22). A meta-analysis by Chen et al. of 91 reports involving 11,886 SOT recipients supports this finding of booster vaccination enhancing immunogenicity in SOT patients (23). Although the subject has not experienced documented COVID-19 illness, and no response was elicited against NP antigens, a degree of reactivity was seen against M protein. It is plausible that this pattern of reactivity reflects a pre-existing immunity induced by prior exposures to the related non-SARS hCoVs. However, it is unclear whether this T cell response will prove durable over many months: In one study of SOT recipients, no immune response was detectable after booster (24). Many SOT patients did not demonstrate robust production of antibodies was not produced until after third dose of mRNA vaccine (25–27), and antibody titers have been shown to wane after three months following a third dose (6). We observed waning of neutralizing antibody after peaking about one-month post third dose of vaccine. Full vaccination has been associated with reduced mortality from COVID-19 illness in SOT patients (28), but these patients may require monitoring for a sustained immune response.

The emergence of SARS-CoV-2 variants, especially omicron BA.4 and BA.5, raises concerns of immune escape like for humoral immunity. Our subject showed partial immune response to variants despite immune escape. We challenged ancestral spike specific T cells from all three time points with seven SARS-CoV-2 variants and noted possible protection against future variants. Most variants showed reactivity/response against WT- and mutant epitope, e.g., against beta variant post booster dose (lost in Draw 3, 13 months), gamma variant (Draw 3, 13 months) and delta variant (Draw 3, 13 months). T cells expanded using ancestral or omicron S1/S2 showed cross-reactivity, suggesting possible protective immune response against omicron variant post vaccination. A robust T-cell response was recognized against several α-hCoV antigens, whereas minimal reactivity was observed against both β-hCoVs; comparable reactivity was observed against novel VOCs and VOIs; Thus, the broad T cell immunity against SARS CoV-2 targets full-length immunodominant viral proteins and might provide partial cross-protection against emerging variants.

Record of immune response is important because full vaccination may not provide permanent protection against COVID-19 illness. Even maximally effective mRNA vaccines (29) may not confer durable immune response against variants of the virus (30, 31). Vaccines may have only moderate effectiveness against the omicron variant and subvariants in both immunocompromised and healthy people (32–35), so this finding is concerning. The bivalent vaccine has proven effective against novel omicron subvariants (36, 37), but this patient’s immune response was measured prior to the introduction of the bivalent vaccine. The immune response may persist even after repeated re-infection, as observed with other non-systemic respiratory viruses like influenza (30). Hybrid immunity (combined immunity from vaccination and breakthrough infections) may be more protective than protection from vaccination alone, but is not yet well characterized (37–39). As new variants of SARS-CoV-2 and novel mechanisms of immune system evasion are discovered (1), such characterizations will prove critical to understanding vaccine effectiveness in immunocompromised patients and in protecting them against COVID-19 illness.

This study had several strengths. The health care worker had an unusual medical history and provided frequent blood samples, allowing measurement of immune response over time with sensitive, specialized assays. We tested the anti-SARS-CoV-2 serum antibody level every 1-2 months from February 2021 to April 2022, when multiple VOCs were described. We performed neutralizing antibody assays with authentic SARS-CoV-2 virus, then measured T-cell response at multiple time points post vaccination and booster shot with large, overlapping peptide libraries spanning the full length of S1, S2, M and NP antigens. The viral samples and libraries detected pre-established T cell responses against viral antigens unavailable to most researchers. These combined strengths contribute to our understanding of this subject’s immune response.

This study also had several limitations. The variant pools in our peptide library consisted of limited epitopes spanning divergent regions. Our results may represent a minor portion of the overall T cell immunity against the full-length spike proteins, versus total anti-spike T cell reactivity induced by vaccination. Second, the cultures in which reciprocal cross-reactivity with Omicron S1/S2-induced T cells were observed did not show a statistically significant difference in the percentage of cross-reactive cells among total reactive T cells. Third, not all antigens tested mounted robust T-cell responses. No response was elicited against NP antigens compared to the reactivity against M protein. This pattern of reactivity may reflect a pre-existing immunity induced by prior exposures to the related non-SARS hCoVs.

In summary, an immunocompromised woman status post SOTs had poor initial humoral response to the COVID-19 mRNA vaccine, then demonstrated a robust, sustained immune response against S1 and S2 antigens for over seven months after receiving the recommended vaccine doses. Her immune response to vaccination indicates partial or more effective formation of anti-spike T cell memory despite chronic immunosuppression, though the full significance of these T cell changes is not yet known. As vaccine effectiveness data is updated, and as better understanding of immune response including hybrid immunity emerges, these findings may reassure that recipients of SOTs may be capable of durable immune responses to emerging variants of SARS-CoV-2.

## 6 Patient perspective

The patient provided written informed consent for this publication and described her treatment as follows:

I am a 63-year-old woman, full time health care worker, whose primary diagnosis is sporadic lymphangioleiomyomatosis (LAM) who required double lung transplantation March 8, 2006 and since then on chronic stable immune suppression including oral tacrolimus (stable plasma levels 5-7 checked monthly), Prednisone 5 mg/day, mycophenolate mofetil 250 mg po twice daily. My complications of lung transplantation include donor transmitted CMV infection December 2006 treated with gancyclovir and foscarnet with resolution, tacrolimus nephrotoxicity requiring related living donor kidney transplant 4/3/15. I was treated with 3 courses of rabbit antithymocyte globulin March 2006, May 2015 (post transplants) and November 2017 (for routine lung biopsy proven antibody mediated lung rejection). Pulmonary function has remained stable and robust with FEV1 >100%, renal function measured as normal with serum creatine 0.77-0.82. I received COVID-19 vaccinations 12/28/2020, 01/14/2021 and boosters 8/24/2021 with bivalent COVID-19 booster 12/1/2022. I had no adverse reactions to the first 3 shots but developed fever and myalgias after the bivalent booster. I received passive SARS-CoV-2 antibody therapy with cilgavimab 300mg/tixagevimab 300mg IM on 4/29/22 as recommended pre-exposure prophylaxis for immune suppressed organ transplant recipients (standard of care as of April 2022). Since the COVID-19 pandemic began, I mask outside my home and I have been well and free from SARS-CoV-2 infection. Infections over the course of the last 2 years include prolonged norovirus gastroenteritis in March-April 2022 and asymptomatic low level EBV viremia noted on routine monitoring June 2022.

## Data availability statement

Due to patient confidentiality and participant privacy the dataset will be available upon request with permission of the third party.

## Ethics statement

The studies involving human participants were reviewed and approved by Columbia University, NIH Clinical Center. The patients/participants provided their written informed consent to participate in this study. Written informed consent was obtained from the individual(s) for the publication of any potentially identifiable images or data included in this article.

## Author contributions

VD, CC-C, and JL contributed to conception and design of the study. JL and MS organized the database. MS and PM performed the flow cytometry and statistical analysis. VD, JL, and MM wrote the first draft of the manuscript. CC-C, MS, and PM wrote sections of the manuscript. All authors contributed to the article and approved the submitted version.
