# Supplementary material for: Case Report: Kinetics and durability of humoral and cellular response of SARS-CoV-2 messenger RNA vaccine in a lung and kidney transplant recipient
Source: Front Immunol. 2023 Jul 3;14:1207638. doi: 10.3389/fimmu.2023.1207638 (PMC10350526; doi:10.3389/fimmu.2023.1207638)
Supplement: Supplementary file 1 [file Presentation_1.pptx]

## Slide 1
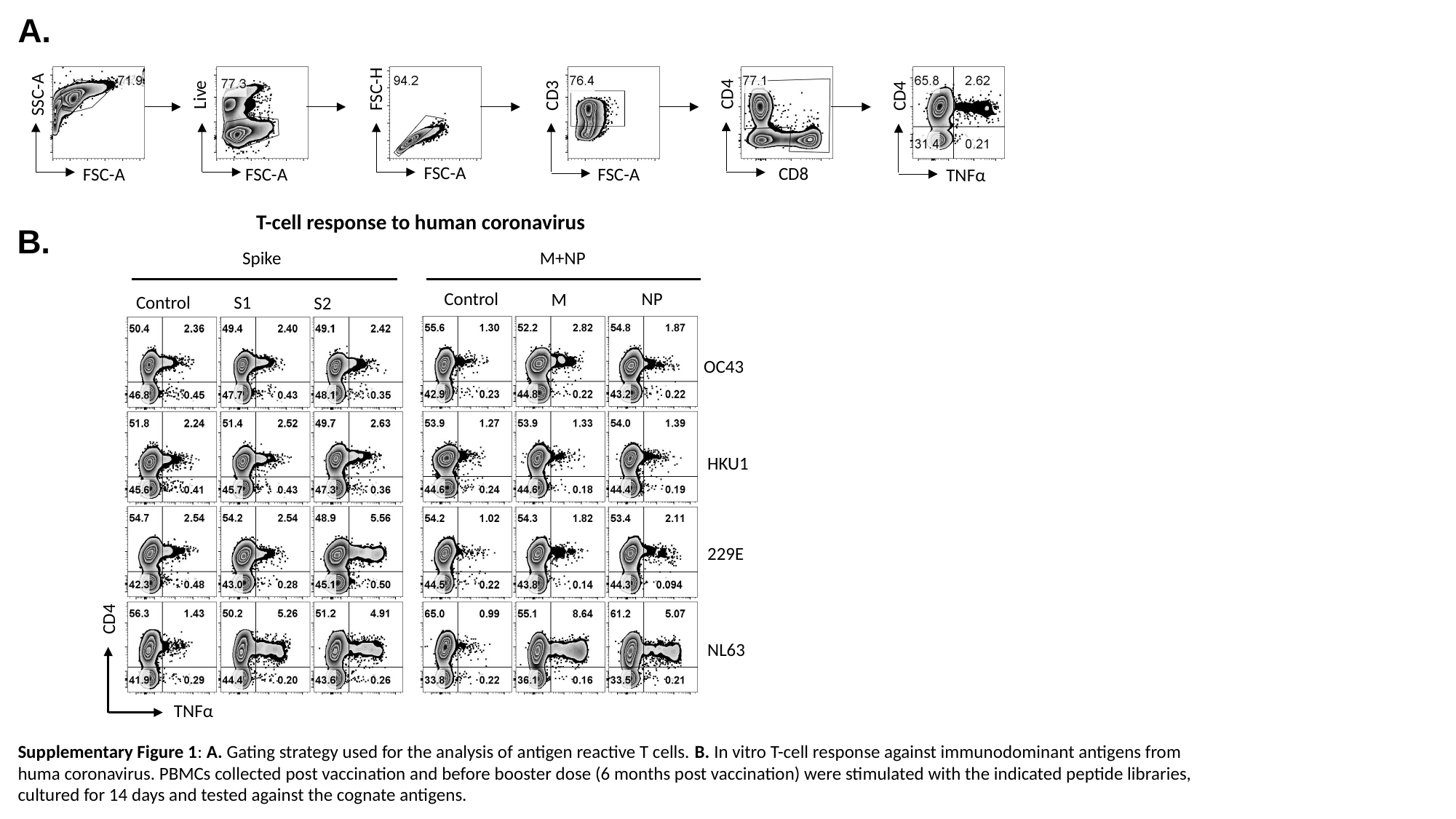

A.
FSC-H
CD4
SSC-A
Live
CD3
CD4
FSC-A
CD8
FSC-A
FSC-A
FSC-A
TNFα
T-cell response to human coronavirus
B.
Spike
M+NP
Control
NP
M
Control
S1
S2
OC43
HKU1
229E
CD4
NL63
TNFα
Supplementary Figure 1: A. Gating strategy used for the analysis of antigen reactive T cells. B. In vitro T-cell response against immunodominant antigens from huma coronavirus. PBMCs collected post vaccination and before booster dose (6 months post vaccination) were stimulated with the indicated peptide libraries, cultured for 14 days and tested against the cognate antigens.
