# Supplementary material for: Case Report: Kinetics and durability of humoral and cellular response of SARS-CoV-2 messenger RNA vaccine in a lung and kidney transplant recipient
Source: Front Immunol. 2023 Jul 3;14:1207638. doi: 10.3389/fimmu.2023.1207638 (PMC10350526; doi:10.3389/fimmu.2023.1207638)
Supplement: Supplementary file 2 [file Presentation_2.pptx]

## Slide 1
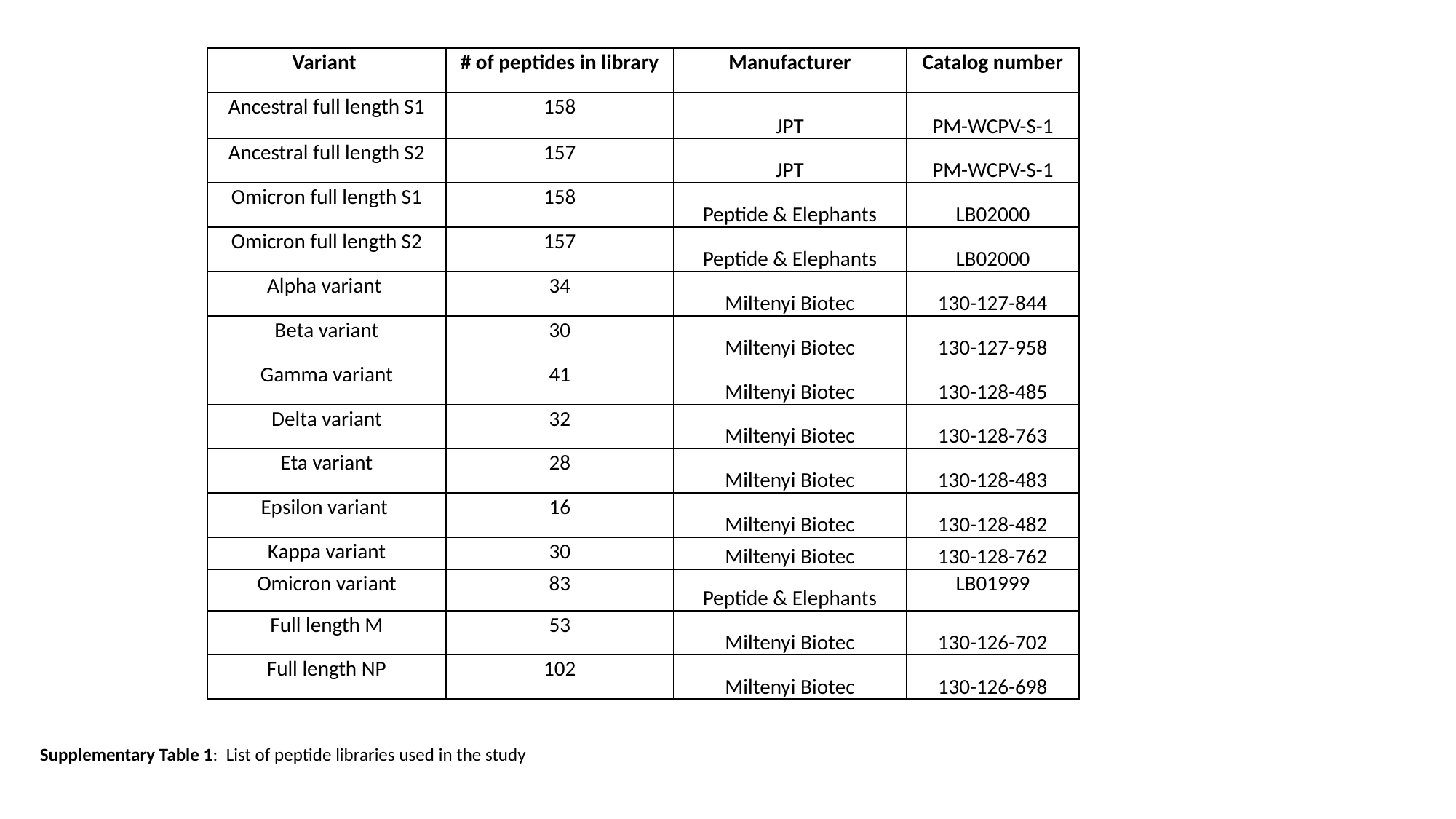

| Variant | # of peptides in library | Manufacturer | Catalog number |
| --- | --- | --- | --- |
| Ancestral full length S1 | 158 | JPT | PM-WCPV-S-1 |
| Ancestral full length S2 | 157 | JPT | PM-WCPV-S-1 |
| Omicron full length S1 | 158 | Peptide & Elephants | LB02000 |
| Omicron full length S2 | 157 | Peptide & Elephants | LB02000 |
| Alpha variant | 34 | Miltenyi Biotec | 130-127-844 |
| Beta variant | 30 | Miltenyi Biotec | 130-127-958 |
| Gamma variant | 41 | Miltenyi Biotec | 130-128-485 |
| Delta variant | 32 | Miltenyi Biotec | 130-128-763 |
| Eta variant | 28 | Miltenyi Biotec | 130-128-483 |
| Epsilon variant | 16 | Miltenyi Biotec | 130-128-482 |
| Kappa variant | 30 | Miltenyi Biotec | 130-128-762 |
| Omicron variant | 83 | Peptide & Elephants | LB01999 |
| Full length M | 53 | Miltenyi Biotec | 130-126-702 |
| Full length NP | 102 | Miltenyi Biotec | 130-126-698 |
Supplementary Table 1: List of peptide libraries used in the study
